# Estimating cancer incidence in Uganda: a feasibility study for periodic cancer surveillance research in resource limited settings

**DOI:** 10.1186/s12885-023-11124-6

**Published:** 2023-08-18

**Authors:** Annet Nakaganda, Angela Spencer, Jackson Orem, Collins Mpamani, Henry Wabinga, Sarah Nambooze, Gertrude N. Kiwanuka, Raymond Atwine, Isla Gemmell, Andrew Jones, Arpana Verma

**Affiliations:** 1 Uganda Cancer Institute, Kampala, Uganda; 2 Department of Public Health and Manchester Academic Health Sciences Centre, University of Manchester, Manchester, UK; 3 Kampala Cancer Registry, Kampala, Uganda; 4 Makerere University College of Health Sciences, Kampala, Uganda; 5 Mbarara University of Science and Technology, Mbarara, Uganda

**Keywords:** Cancer, Incidence, Registration, Surveillance

## Abstract

**Background:**

Population based cancer registries (PBCRs) are accepted as the gold standard for estimating cancer incidence in any population. However, only 15% of the world’s population is covered by high quality cancer registries with coverage as low as 1.9% in settings such as Africa. This study was conducted to assess the operational feasibility of estimating cancer incidence using a retrospective “catchment population” approach in Uganda.

**Methods:**

A retrospective population study was conducted in 2018 to identify all newly diagnosed cancer cases between 2013 and 2017 in Mbarara district. Data were extracted from the medical records of health facilities within Mbarara and from national and regional centres that provide cancer care services. Cases were coded according to the International Classification of Diseases for Oncology (ICD-0-03). Data was analysed using CanReg5 and Excel.

**Results:**

We sought to collect data from 30 health facilities serving Mbarara district, southwestern Uganda. Twenty-eight sources (93%) provided approval within the set period of two months. Among the twenty-eight sources, two were excluded, as they did not record addresses for cancer cases, leaving 26 sources (87%) valid for data collection. While 13% of the sources charged a fee, ranging from $30 to $100, administrative clearance and approval was at no cost in most (87%) data sources. This study registered 1,258 new cancer cases in Mbarara district. Of the registered cases, 65.4% had a morphologically verified diagnosis indicating relatively good quality of data. The Age-Standardised Incidence Rates for all cancers combined were 109.9 and 91.9 per 100,000 in males and females, respectively. In males, the most commonly diagnosed cancers were prostate, oesophagus, stomach, Kaposi’s sarcoma and liver. In females, the most common malignancies were cervix uteri, breast, stomach, liver and ovary. Approximately, 1 in 8 males and 1 in 10 females would develop cancer in Mbarara before the age of 75 years.

**Conclusion:**

Estimating cancer incidence using a retrospective cohort design and a “catchment population approach” is feasible in Uganda. Periodic studies using this approach are potentially a precious resource for producing quality cancer data in settings where PBCRs are scarce. This could supplement PBCR data to provide a detailed and comprehensive picture of the cancer burden over time, facilitating the direction of cancer control efforts in resource-limited countries.

## Background

Cancer is the leading cause of death in developed countries and second to cardiovascular diseases in developing countries [1]. Cancer control requires accurate estimates of the cancer burden, to enable rational planning and monitoring of cancer control programmes [2, 3]. Accurate estimates of the cancer burden are mainly provided by Population Based Cancer Registry (PBCR) data; and PBCRs are accepted as the gold standard for estimating the cancer burden in any population [1, 2, 4]. However, only 15% of the world population is covered by high quality cancer registries, with a lower coverage in settings such as Africa (1.9%) [1, 3]. In settings where such registries do not exist, the cancer burden is estimated using statistical modelling techniques, based on data from regional registries or neighbouring countries [3, 5].

In Uganda, the cancer burden estimates are mainly based on one population based cancer registry, Kampala Cancer Registry [6]. This registry covers about 8% of the total population and is urban based, while 80% of the Ugandan population is rural [7–9]. Consequently, the cancer burden and variation in cancer occurrence among different regions of the country are not known and there is a lack of detailed descriptive epidemiology of cancer in other regions [6]. One way to address this is to establish other regional population based cancer registries that will permit assessment of cancer occurrence and variations in these regions and contribute to the aggregated estimation of the national cancer burden [9]. However, developing and maintaining high quality PBCRs is still not feasible for many resource-limited countries like Uganda. As a result, resource-limited settings lack empirical local data on the cancer burden for planning and implementing evidence based cancer control strategies [10].

The primary objective of this study was to assess the operational feasibility (accessibility and acceptability of data sources; availability of cancer data to estimate cancer incidence; and quality of data) of estimating cancer incidence using a catchment population approach in Mbarara district (a district not covered by PBCRs in Uganda). The second objective was to estimate cancer incidence in Mbarara district and to document the resources that might be used to conduct such a study in resource limited settings. If successful, this method could be applied to those regions for which the development and maintenance of population-based cancer registries is not currently possible and would ultimately allow evaluations of cancer control interventions at population level, to inform and direct cancer control policies at all levels.

## Methods

### Study population

A retrospective population study based on a “catchment population approach” was conducted in October 2018 –January 2019. A “catchment population approach” involves counting the number of newly diagnosed cases (based on routine clinical care and diagnosis) in all the health facilities serving a defined population [11]. Hence, this study registered all newly diagnosed cancer cases, in the period of 2013 to 2017, in Mbarara district. Mbarara District is located in South Western Uganda, about 290 km (by road) from the capital city of Kampala. Mbarara municipality is the largest city in the sub-region, with 59% of its population living in the city[12]. Subsistence farming employs more than half of the population. In comparison to the national average of 20%, 27% of people in Mbarara district live below the national poverty line [12, 13].

### Data sources and method of data collection

The registration of cancer cases involved extracting data, using standardised Data Abstraction Forms (DAF), from medical records of all health facilities within Mbarara district. Data were also collected from national and regional centres that provide cancer diagnosis, treatment and palliative care services. The data sought included: socio-demographic characteristics (age, sex, date of birth, occupation, marital status, tribe/ethnicity, residence and religion); tumour characteristics, (incidence date, basis of diagnosis, topography, morphology, behaviour of the tumour); and treatment and follow-up (stage of disease, initial treatment and status of last contact). We aimed to collect information on selected cancer risk factors including: history of alcohol use; tobacco use; physical inactivity; overweight and obesity; unhealthy feeding; and reproductive factors. In addition, we collected data on relevant infections such as HIV, HPV, Hepatitis B/C and Helicobacter pylori.

The research team comprised seven people with a minimum work experience of more than five years in cancer research and cancer registration. This team was trained using both; on-line training for human subject protection, and two-day face-to-face training. Topics covered were: team work; research ethics; introduction to Good Clinical Practice (GCP); documentation in research; background, rationale and methodology of the research protocol; standard operating procedures of the study; data management; and quality assurance and control procedures.

A “cancer case” was defined as any cancer patient with a diagnosis based on: either clinical history alone, or clinical history with other investigations such as x-ray, microscopic, cytology, autopsy, histology (microscopic) or immunohistochemistry. This case definition is in line with the general guidelines for cancer surveillance set by the International Agency for Research on Cancer (IARC) [14]. Cases were excluded if their date of diagnosis was outside the study period (2013–2017) and/or if the patient was not a resident in Mbarara district. The abstracted cancer cases (topography and morphology) were coded according to the third edition of the International Classification of Diseases for Oncology (ICD-0-03).

Quality of data was assured by use of Standard Operation Procedures (SOPs) and a Data Quality Management Plan (DQMP). SOPs and DQMP were developed based on international handbooks and guidelines from International Agency for Research on Cancer (IARC), African Cancer Registry Network (AFCRN), the International Association of Cancer Registries (IACR) and the Union for International Cancer Control (UICC) [126, 148]. The SOP and DQMP standardized the definition, collection and recording of study variables: date of birth, age, sex, residence address, date of incidence, most valid basis of diagnosis, site of the primary, multiple primaries, morphology, and behavior of the tumour.

Special measures for monitoring and assuring the quality of the data abstraction process were employed including training of study staff in quality control measures; real-time review and audit of abstracted forms; data entry checks and generation of weekly data queries; holding weekly data cleaning and error correction meetings; interim analysis of the records (593 records) during data collection; and final data cleaning and consistence checks by the investigator before data analysis. Data quality was assessed using three parameters: (1) proportion of cases with missing data; (2) independent double data extraction for a random sample of records and assessing level of agreement between abstractors; and (3) percentage of morphologically verified cases.

The proportion of cases with unknown or missing variables was based on the mandatory variables required for each cancer case, as determined by IARC [14]. There are mandatory variables to be recorded on each case and it is not acceptable to have some variables, such as sex, missing. Other variables, like age, have an acceptable maximum percentage (< 20%) of cases with unknown or missing variables, which was set by CI5 volume IX [15]. Similarly, the proportion of cases with morphologically verified diagnosis (MV %) refers to the percentage of cases for which diagnosis is based on histology or cytology, and a higher MV% represents a greater accuracy of diagnosis [15].

Independent double data extraction was done by two different data abstractors in a time interval of four weeks. A sample of records for re-abstraction was selected using systematic sampling, taking every 5th record from number one as in the database. The number of records re-abstracted was 80 records and this number was determined by convenience, according to available resources in terms on money and time (that is, the second abstractor was contracted for two weeks and took the number of records he managed to re-abstract within that period, which was 80 records). The variables that were re-abstracted included; age, sex, tribe, incident date; basis of diagnosis; site of primary; morphology; staging; date of last contact; and status of last contact. The level of agreement was calculated by comparing the agreement between the original abstractor and the re-abstractor’s findings at the data element level (one-to-one comparison). This was done by counting the number of times the original abstractor and re-abstractor agreed on a variable, across all the compared variables, and dividing it by the total number of records being compared (in this case 80 records) and then converting it to a percentage.

### Data management and analysis

Data were managed and analysed using CanReg5 and Excel. CanReg5 software has the ability to detect duplicates and multiple registration of cases (duplicate registration) was minimized by use of multiple and sufficient identifiers/items on the DAF, to ensure recognition of duplicate sources that may come from different sources [16]. Data entrants were trained on identifying duplicates using CanReg5 software, matching of records relating to the same patient from more than one sources and to update the records with new information.

Data analysis calculated the age standardized incidence rates, using World Standard population, for 5-year age groups. Incidence rates were based on the population of Mbarara district for the years of 2013 to 2017. The population of Mbarara district for years 2014 to 2017, by sex and 5-year age groups, was obtained from Uganda Bureau of Statistics (UBOS). Population estimates for 2013 were obtained using simple linear regression trend analysis. In addition, cumulative risk was calculated to estimate the lifetime risk of developing cancer in Mbarara district. Cumulative risk is the probability that individuals in a population will develop a specified form of cancer during a certain age span [8, 17, 18].

### Feasibility measurement criteria

The primary objectives were measured as:

- Accessibility and acceptability of data source was measured as the percentage of health facilities accessed and whose approval was obtained within two months of application;
- Availability of cancer data to estimate cancer incidence was assessed by the availability of mandatory variables for estimating cancer incidence, and the ability to estimate cancer incidence rates using CanReg5;
- Quality of data was measured as the percentage of cases with a morphological diagnosis (above 61%) and missing cases (below 20%), according to standards set by the International Agency for Research on Cancer (IARC) international guidelines.

#### Ethics

The study was reviewed and approved by the University of Manchester and Uganda Cancer Institute Research and Ethics Committees. Further regulatory clearance was obtained from the Uganda National Council for Science and Technology (UNCST).

## Results

### Accessibility and acceptability of the data sources

The process of accessing the study population involved several steps including: mapping of the data sources (health facilities); obtaining administrative approvals from the data sources; presentation of the study protocol to the data sources and making a source-specific plan for data collection; and visiting the data source for actual data collection. Identification and mapping of the data sources (health facilities) was done by the research team, generating a list of data sources that was continuously updated during actual field work, as more health facilities came to the knowledge of the researchers. We sought to collect data from 30 sources (listed and described in Table 1), of which twenty-eight sources (93%) provided approval within the set period of two months. The study found out that the process of accessing the data differs among data sources: While all sources required ethical approval letters of the study, each data source had its own administrative requirements, approval process and timelines for accepting the study. Also, administrative approval to access the medical records was at no cost in most (87%) of the sources, but four sources (13%) charged a fee that ranged from $30 to $100 USD.

Another observation is that there are variations in documentation among health facilities, and hence not all sources were valid for data collection. Among the twenty-eight sources that provided timely approval for data collection two sources were invalid because one does not routinely record places of residence for cancer cases and one was a sample collection centre, and as such did not hold patient diagnostic records. Overall four important sources were excluded for data collection. Excluding these four sources could have impacted the completeness of case-finding, resulting in an underestimation of cancer incidence.

### Characteristics of the data sources

The proportion of the data sources that provided data was 86% (26 out of 30 sources mapped for data collection) and all of them were operational throughout the study period. Among the twenty-six sources that provided data, eleven (42%) were hospitals; six (23%) health centres (HCIV); seven (27%) pathology laboratories; and two (8%) were hospice centres. These data sources were managed and owned by different entities, ranging from government owned health facilities (ten sources), nine privately owned, five faith-based. and two not-for-profit facilities. 54% (n = 14) of the data sources were from the western region and 46% (n = 12) were from the central region, Kampala city. These sources provided various cancer services including: cancer screening; cancer diagnosis; cancer treatment; and palliation and end-of-life care, see Table 1.

**Table 1:** Characteristics of the data sources used to estimate cancer incidence in Mbarara district

Serial #	Name of the Health facility	Level of the facility	Ownership	Location	Cancer services offered at the facility	Included in the study (Yes/No)
1.	Mbarara Regional Referral Hospital	Regional Referral Hospital	Government	Western region-Urban	Screening, diagnosis, treatment	Yes
2.	Holy Innocent Children’s Hospital	Hospital	Faith based-not for profit	Western region-Urban	Screening	Yes
3.	Mbarara Medical/Cancer Center	Health Centre	Private	Western region-Urban	Treatment (Chemotherapy)	Yes
4.	Bugamba HC IV	Health Centre IV	Government	Western region-Rural	Cervical cancer screening	Yes
5.	Bwizibwera HC IV	Health Centre IV	Government	Western region-Rural	Cervical cancer screening	Yes
6.	Kinoni HC IV	Health Centre IV	Government	Western region-Rural	Cervical cancer screening	Yes
7.	Mayanja Memorial Hospital	Hospital	Private	Western region –Urban	Cervical cancer screening	Yes
8.	Mbarara community Hospital	Hospital	Private	Western region Urban	Screening	Yes
9.	Mbarara municipal HC IV	Health IV	Government	Western region-Urban	Cervical cancer screening	Yes
10.	Ruharo Mission Hospital	Hospital	Faith-based	Western region-Urban	Treatment (surgery/chemo)	Yes
11.	UPDF 2nd Division Hospital	Health Centre IV	Government	Western region-Urban	Screening	Yes
12.	Hospice Africa Uganda-Mbarara	Hospice-	Private-not-for profit	Western region-Urban	Palliation	Yes
13.	Multisystems Lab	Laboratory	Private	Central region-Urban	Diagnostics	Yes
14.	Metromed Lab	Laboratory	Private	Central region-Urban	Diagnostics	Yes
15.	Makerere Pathology Lab	Laboratory	Government	Central region-Urban	Diagnostics	Yes
16.	Makerere pathology Lab (Core)	Laboratory	Private	Central region-Urban	Diagnostics	Yes
17.	Hospice Africa Uganda-Kampala	Hospice-	Private-not-for profit	Central region-Urban	Palliation	Yes
18.	Mengo Hospital	Hospital	Faith-based	Central region -Urban	Screening, treatment-surgery	Yes
19.	Nsambya Hospital	Hospital	Faith-based	Central region -Urban	Screening, treatment	Yes
20.	Path diagnostics	Laboratory	Private	Central region -Urban	Diagnostics	Yes
21.	Rubaga Hospital	Hospital	Faith-based	Central region -Urban	Screening, surgery treatment	Yes
22.	Mulago Hospital	National Referral Hospital	Government	Central region -Urban	Screening, diagnosis, treatment	Yes
23.	Uganda Cancer Institute	National cancer Hospital	Government	Central region -Urban	All cancer services	Yes
24.	Surg-path Lab	Laboratory	Private	Central region-Urban	Diagnostics	Yes
25.	Divine Mercy Hospital	Hospital	Private	Western region-Urban	Treatment (surgery)	Yes
26.	MUST Pathology Lab in Mbarara*	Laboratory	Government	Western region -Urban	Diagnostics	Yes
27.	NIRA*	Death registration office	Government	Central region- Urban	Death registration	No (no approval yet)
28.	Lancet Laboratories	Laboratory	Private	Central region-Urban	Diagnostics	No (no approval yet)
29.	LMK* Lab	Laboratory	Private	Central region-Urban	Diagnostics	No (lacks residential addresses)
30.	MBN* laboratory	Laboratory	Private	Central region-Urban	Diagnostics	No (sample collection centre only)

NIRA = National Identification and Registration Authority; MUST = Mbarara University of Science and Technology; MBN & LMK are full names

### Availability of cancer data to estimate cancer incidence

#### Characteristics of the cancer cases

Table 2 provides selected socio-demographic characteristics of the cancer cases. During the five years considered, 2013 to 2017, 1258 cancer cases were recorded: 656 females and 602 males. Of these, 5.2% (65) were children aged below 15 years. Among the cases, the most represented ethnic groups were Banyankore (83.4%) who represent the largest population in this area, followed by Baganda (4.8%).

**Table 2:** Socio-demographic characteristics of cancer cases, Mbarara district

Variable	Frequency (n = 1258)	Percentage (%)
**Age group (years)**		
≤ 14	65	5.2
15–29	118	9.4
30–44	259	20.6
45–59	331	26.3
60–74	289	23.0
≥ 75	196	15.6
**Sex at birth**		
Male	602	47.9
Female	656	52.1
**Tribe/Ethnic group**		
Banyankore	1049	83.4
Baganda	60	4.8
Bakiga	40	3.2
Banyarwanda	35	2.8
Others	74	5.9
**Occupation**		
Peasant	218	17.3
Elementary occupations	148	11.8
Service and sales workers	109	8.6
Professionals	63	5.0
Plants/machine operators/assemblers	68	5.4
Unknown/Not Applicable	471	37.4
Other (armed forces, students, crafts, agriculture, forestry, fishery)	181	14.4
**Religion**		
Christian	884	70.3
Muslim	34	2.7
Other	340	27.0

**Fig. 1:**
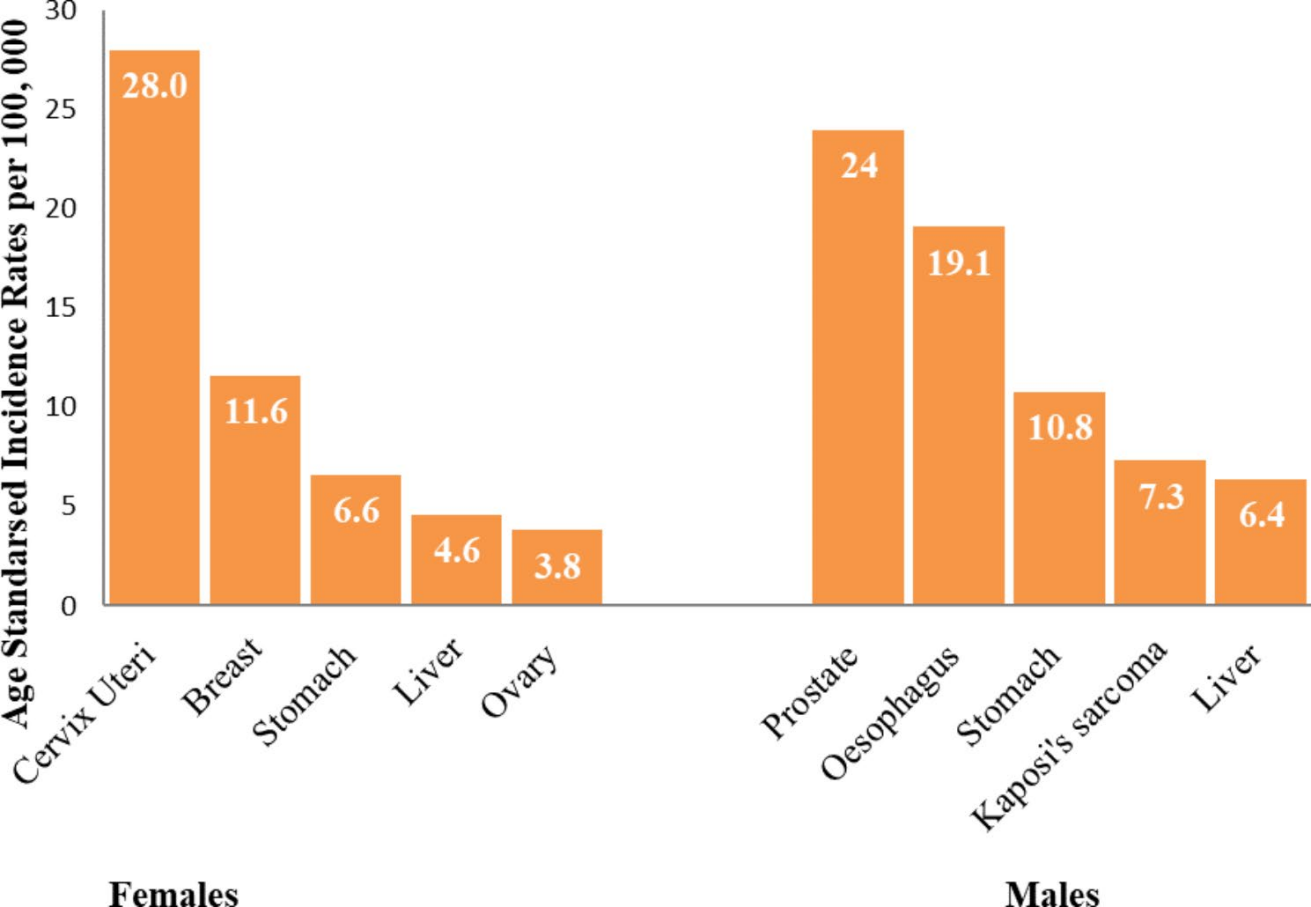
Incidence for the top five cancers, by sex, Mbarara district, 2013–2017

**Estimating cancer incidence in Mbarara district**: Fig. 1 shows the incidence rates for the top five cancers in females and males, and Table 3 shows the incidence rates for all the cancers identified in this study. The Age-Standardised cancer incidence rates (ASRs), for all cancers combined, in Mbarara district were 109.8 per 100,000 in males (95% CI: 98.2-117.5) and 91.9 per 100,000 in females (95% CI: 82.5–97.7). Overall, both sexes combined, cervical cancer was the most commonly diagnosed cancer, followed by prostate, oesophagus, breast and stomach. By sex, the most commonly diagnosed cancers in males, over the study period, were prostate, oesophagus, stomach, Kaposi’s sarcoma and liver. In females, the most common malignancies were cervix uteri, breast, stomach, liver and ovary.

**Table 3:** Incidence for all cancers identified in Mbarara district, (2013–2017)

	Male						Female							
Site	No. cases	Freq. (%)	Crude rate	ASR	Cum. Rates (%)		No. cases	Freq. (%)	Crude rate	ASR	Cum. Rates (%)		ICD-10	
			(Per 100,000)		0–64	0–74			(Per 100,000)		0–64	0–74		
Tongue	9	1.5	0.8	**1.6**	0.17	0.36	3	0.5	0.2	**0.6**	0.04	0.04	C01-02	
Mouth	11	1.9	0.9	**1.9**	0.11	0.17	2	0.3	0.2	**0.2**	0.00	0.04	C03-06	
Salivary glands	4	0.7	0.3	**0.8**	0.07	0.07	1	0.2	0.1	**0.1**	0.01	0.01	C07-08	
Tonsil	1	0.2	0.1	**0.2**	0.00	0.00	0	0.0	0.0	**0.0**	0.00	0.00	C09	
Other oropharynx	2	0.3	0.2	**0.1**	0.01	0.01	0	0.0	0.0	**0.0**	0.0	0.0	C10	
Nasopharynx	4	0.7	0.3	**0.5**	0.04	0.04	6	0.9	0.5	**0.6**	0.06	0.06	C11	
Hypopharynx	1	0.2	0.1	**0.3**	0.00	0.06	1	0.2	0.1	**0.2**	0.00	0.04	C12-13	
Pharynx unspecified	1	0.2	0.1	**0.1**	0.00	0.00	1	0.2	0.1	**0.2**	0.00	0.00	C14	
Oesophagus	94	15.9	8.0	**19.1**	1.16	2.14	23	3.6	1.9	**3.8**	0.32	0.44	C15	
Stomach	49	8.3	4.2	**10.8**	0.74	1.40	42	6.5	3.4	**6.6**	0.36	0.79	C16	
Colon	12	2.0	1.0	**2.6**	0.17	0.36	17	2.6	1.4	**2.7**	0.17	0.34	C18	
Rectum	6	1.0	0.5	**1.2**	0.12	0.12	15	2.3	1.2	**2.4**	0.17	0.33	C19-20	
Anus	5	0.8	0.4	**1.0**	0.07	0.14	2	0.3	0.2	**0.2**	0.01	0.01	C21	
Liver	32	5.4	2.7	**6.4**	0.44	0.81	32	5.0	2.6	**4.6**	0.34	0.42	C22	
Gallbladder etc.	0	0.0	0.0	**0.0**	0.00	0.00	4	0.6	0.3	**0.7**	0.03	0.12	C23-24	
Pancreas	8	1.4	0.7	**1.7**	0.09	0.21	5	0.8	0.4	**0.6**	0.03	0.03	C25	
Nose, sinuses etc.	1	0.2	0.1	**0.1**	0.00	0.00	2	0.3	0.2	**0.3**	0.03	0.03	C30-31	
Larynx	13	2.2	1.1	**3.6**	0.12	0.41	1	0.2	0.1	**0.2**	0.03	0.03	C32	
Trachea, bronchus and lung	16	2.7	1.4	**3.0**	0.18	0.30	12	1.9	1.0	**1.9**	0.13	0.17	C33-34	
Other thoracic organs	1	0.2	0.1	**0.1**	0.01	0.01	0	0.0	0.0	**0.0**	0.00	0.00	C37-38	
Bone	3	0.5	0.3	**0.3**	0.02	0.02	6	0.9	0.5	**0.4**	0.02	0.02	C40-41	
Melanoma of skin	1	0.2	0.1	**0.2**	0.02	0.02	5	0.8	0.4	**0.6**	0.01	0.09	C43	
Other skin	8	1.4	0.7	**1.4**	0.13	0.13	9	1.4	0.7	**1.3**	0.12	0.12	C44	
Kaposi sarcoma	65	11.0	5.5	**7.3**	0.45	0.72	22	3.4	1.8	**2.3**	0.16	0.21	C46	
Connective and soft tissue	6	1.0	0.5	**0.6**	0.05	0.05	7	1.1	0.6	**0.7**	0.06	0.06	C47, 49	
Breast	9	1.5	0.8	**1.7**	0.06	0.21	79	12.2	6.4	**11.6**	0.95	1.23	C50	
Vulva							9	1.4	0.7	**1.2**	0.08	0.13	C51	
Vagina							2	0.3	0.2	**0.2**	0.01	0.01	C52	
Cervix uteri							187	28.9	15.1	**28.0**	2.27	2.98	C53	
Corpus uteri							8	1.2	0.6	**1.3**	0.06	0.15	C54	
Uterus unspecified							10	1.5	0.8	**1.3**	0.12	0.12	C55	
Ovary							27	4.2	2.2	**3.8**	0.33	0.37	C56	
Other female genital organs							1	0.2	0.1	**0.2**	0.02	0.02	C57	
Penis	19	3.2	1.6	**3.6**	0.28	0.42								
Prostate	110	18.6	9.4	**24.0**	0.36	2.89								
Testis	3	0.5	0.3	**0.3**	0.02	0.02								
Kidney	6	1.0	0.5	**0.4**	0.02	0.02	7	1.1	0.6	**0.8**	0.01	0.10	C64	
Renal Pelvis	0	0.0	0.0	**0.0**	0.00	0.00	1	0.2	0.1	**0.1**	0.01	0.01	C65	
Ureter	1	0.2	0.1	**0.2**	0.02	0.02	0	0	0.0	**0.0**	0.00	0.00	C66	
Bladder	1	0.2	0.1	**0.3**	0.04	0.04	5	0.8	0.4	**0.4**	0.02	0.02	C67	
Other urinary organs	0	0.0	0.0	**0.0**	0.00	0.00	1	0.2	0.1	**0.1**	0.00	0.00	C68	
Eye	17	2.9	1.4	**1.9**	0.16	0.16	26	4.0	2.1	**2.2**	0.12	0.20	C69	
Brain, nervous system	2	0.3	0.2	**0.6**	0.04	0.10	8	1.2	0.6	**1.2**	0.07	0.12	C70-72	
Thyroid	0	0.0	0.0	**0.0**	0.02	0.02	6	0.9	0.5	**0.7**	0.04	0.08	C73	
Hodgkin disease	13	2.2	1.1	**1.6**	0.11	0.16	11	1.7	0.9	**1.1**	0.06	0.11	C81	
Non-Hodgkin lymphoma	26	4.4	2.2	**3.4**	0.26	0.40	15	2.3	1.2	**1.5**	0.09	0.13	C82-85, C96	
Multiple myeloma	3	0.5	0.3	**0.8**	0.10	0.10	3	0.5	0.2	**0.7**	0.09	0.09	C90	
Lymphoid leukaemia	7	1.2	0.6	**1.0**	0.07	0.07	2	0.3	0.2	**0.1**	0.01	0.01	C91	
Myeloid leukaemia	7	1.2	0.6	**0.9**	0.04	0.10	8	1.2	0.6	**0.8**	0.07	0.07	C92-94	
Leukaemia unspecified	4	0.7	0.3	**0.4**	0.03	0.03	4	0.6	0.3	**0.6**	0.06	0.06	C95	
Other and unspecified	19	3.2	1.6	**4.0**	0.19	0.49	17	2.6	1.4	**2.7**	0.24	0.29	O&U	
*All sites	600		51.1	**109.9**	5.90	12.64	655	-	53.0	**91.9**	6.83	9.68	All	
**All sites but C44	592		50.4	**108.6**	5.77	12.51	646	100.0	52.3	**90.6**	6.71	9.56	AllbC44	

Table produced by CanReg5; ASR = Age standardized rate; Cum. Rate = Cumulative rates;*3 cases with missing age were excluded automatically from the analysis by CanReg5; * * Non-melanoma skin cancer (C44) is often excluded from comparative analyses of cancer data, because of concerns about the completeness of registration (case ascertainment) and a perception that cancers of this kind are rarely life threatening

### Cancer incidence by sex in Mbarara district

**Fig. 2:**
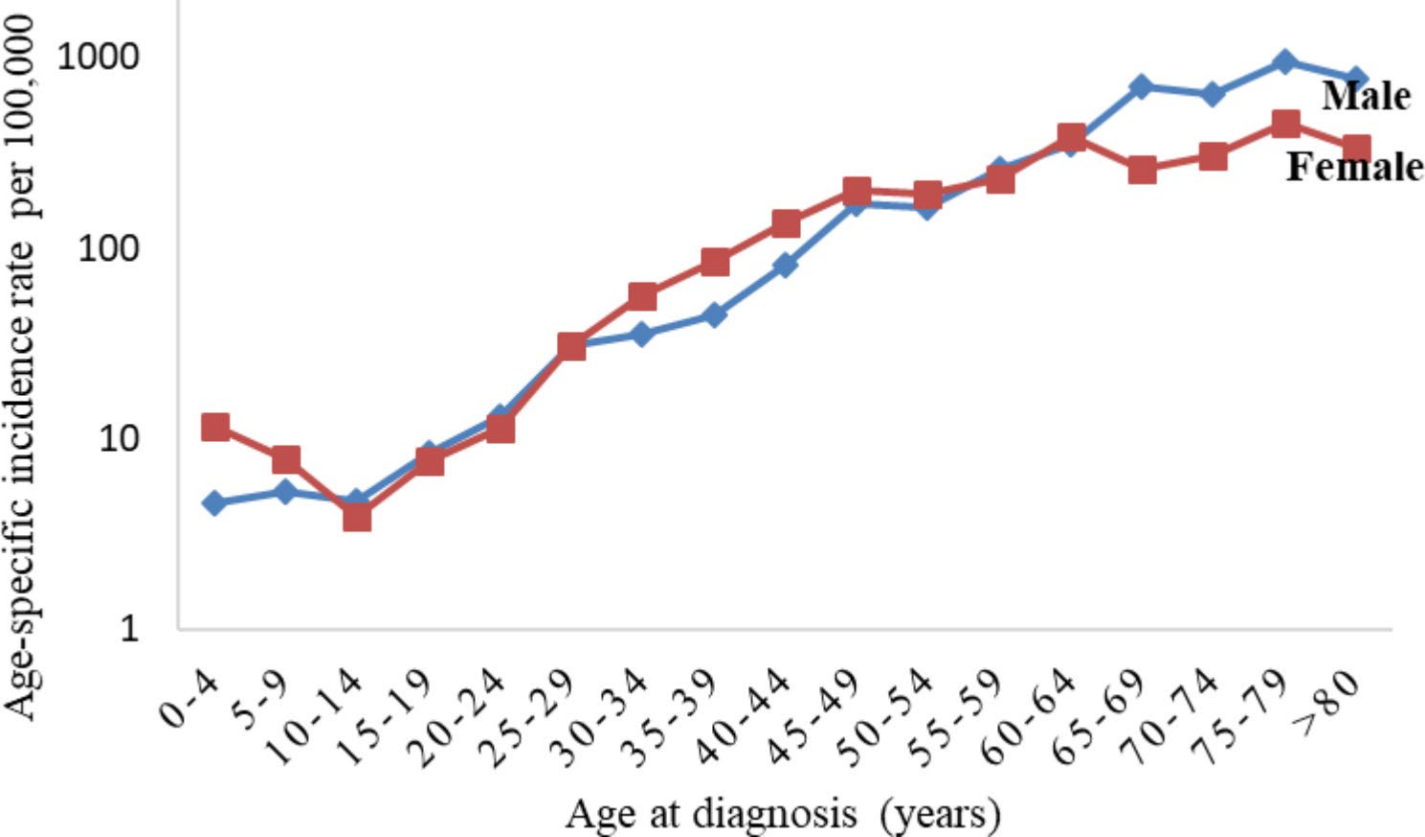
Incidence rates for all cancers combined, by age and sex, Mbarara, 2013–2017

Overall, cancer incidence in Mbarara district was higher in males than in females; 109.9 per 100,000 in males (95% CI: 98.2-117.5) compared to 91.9 per 100,000 in females (95% CI: 82.5–97.7). However, this differs by age group: according to Fig. 2, incidence rates are higher in females in early years of life (0-4-year age group) and in the 30-54-year-olds, but almost the same for both sexes in 5–29 years’ age groups. After the age of 64 years, male incidence rates significantly rise above those of females with its peak at 75-79-years (ASR of 955 per 100,000). For example:

- the incidence rate in females for 0 to 4-year age group is 11.7 per 100,000 (95% CI: 7.2–17.9) compared to 4.6 per 100,000 (95% CI: 2.1–8.7) in males;
- the incidence rate in females for 35 to 39-year age group is 85.1 per 100,000 (95% CI: 62.4-107.8) compared to 45 per 100,000 (95% CI: 29.4–66.2) in males; and.
- the incidence rate in females for 65 to 69-year age group is 259.3 per 100,000 (95% CI: 173.8-373.4) compared to 694 per 100,000 (95% CI: 518.5-869.5) in males.

#### Distribution of cancers by age group in Mbarara district

**Fig. 3:**
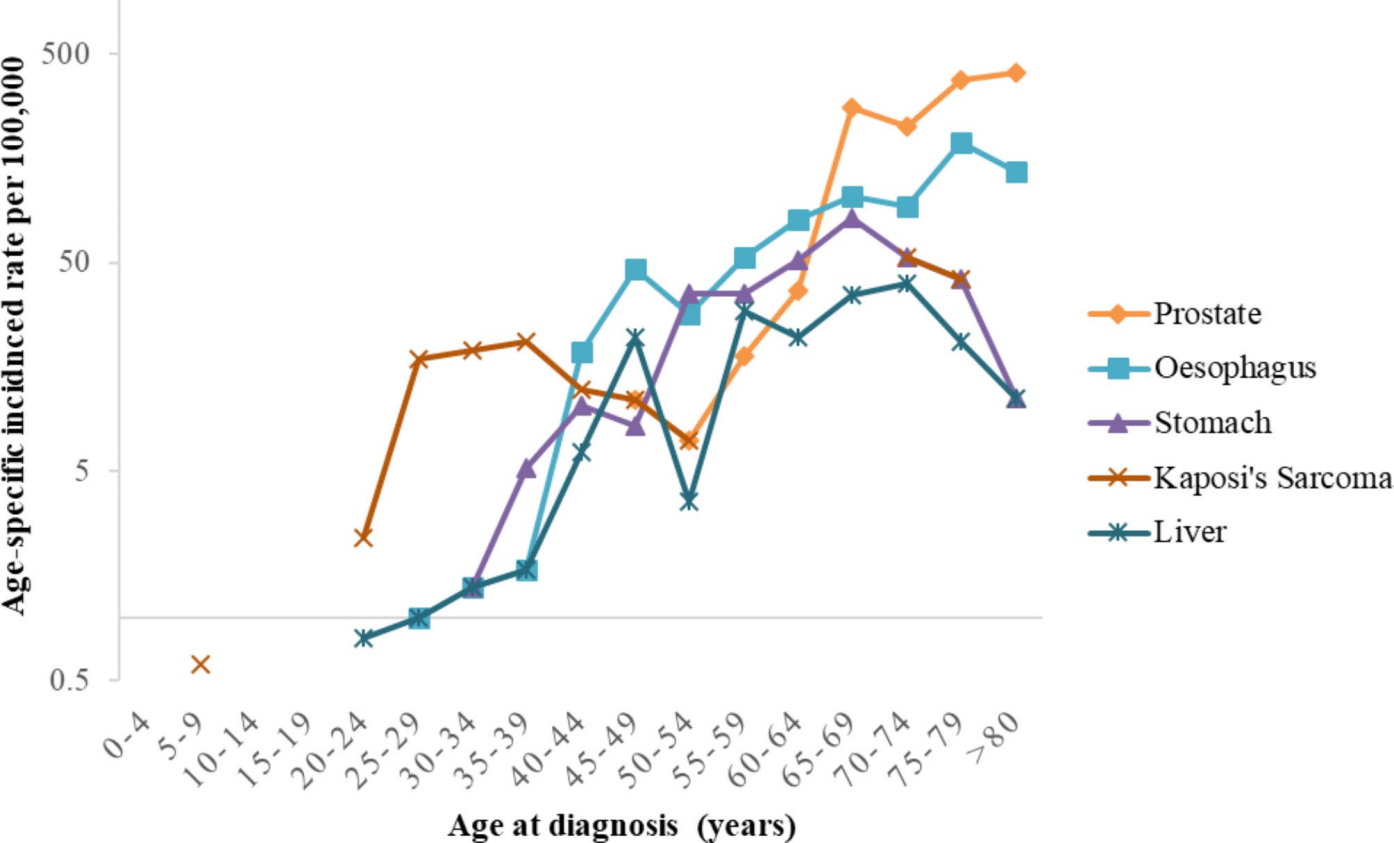
Distribution of top five-cancers, by age, in Males, Mbarara, 2013–2017

**Fig. 4:**
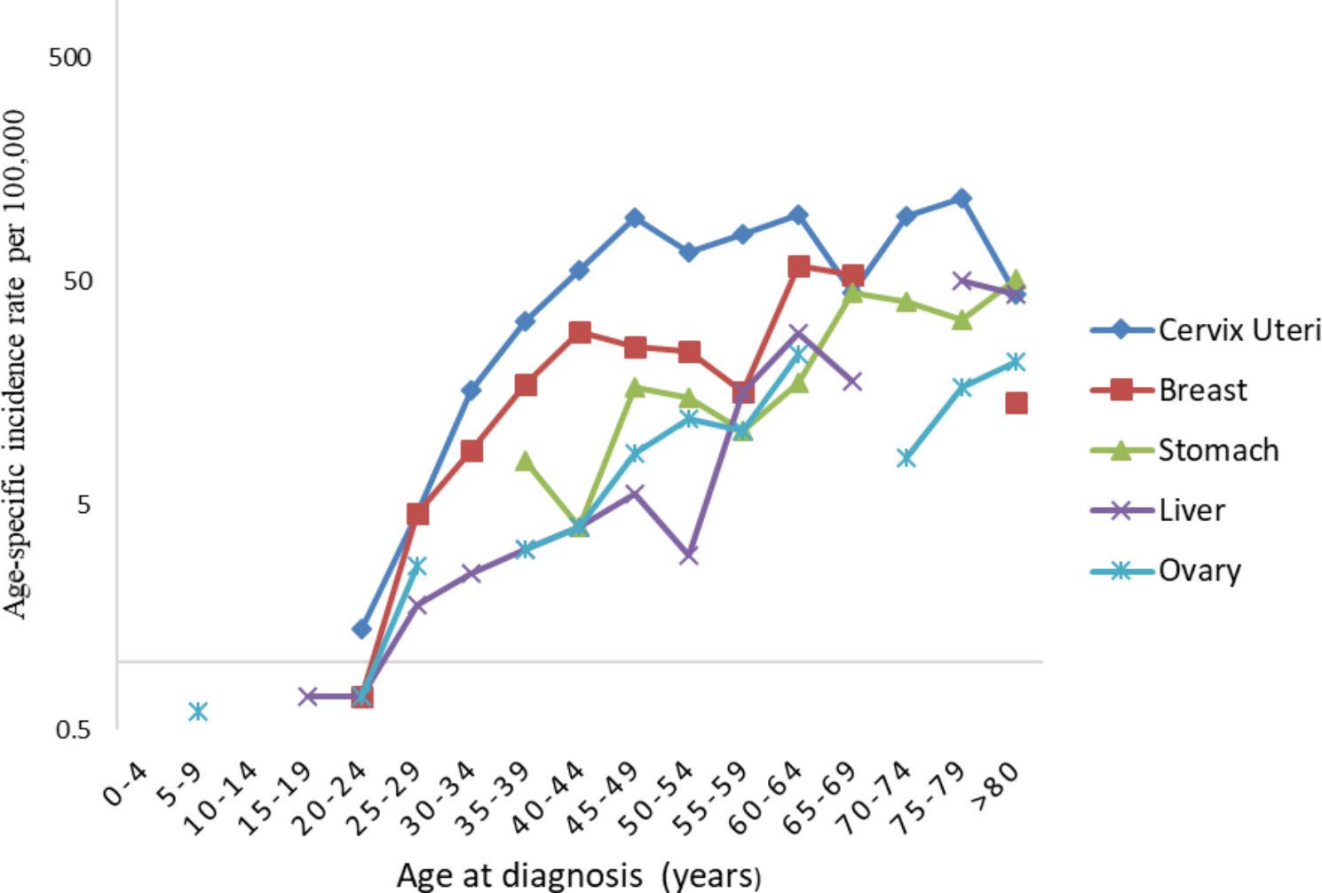
Distribution of top-five cancers, by age, in Females, Mbarara (2013–2017)

Among children below fifteen years, Eye (Retinoblastoma) was the most diagnosed cancer accounting for 16.4%, followed by Kidney (15%), Non-Hodgkin’s lymphoma (15%), and Leukemia (14%). Among males, Kaposi’s sarcoma is the commonest cancer in the 20-39-year age groups; oesophagus highest in the 40-64-year age groups; and prostate commonest in the 65-year and above age groups (Fig. 3). In females, cancer of the cervix is the highest in all the 30-74-year age groups followed by breast and liver: This pattern changes at 75 years where stomach cancer becomes highest, followed by liver and breast cancers, see Fig. 4. The shape of the curves (Figs. 3 and 4) confirms that cancer incidence rates increase with increasing age in both males and females.

##### Lifetime risk of developing cancer

The overall risk of developing any form of cancer within 0 to 74 years’ lifetime, in Mbarara district, is 12.6% in males and 9.7% in females. In other words, approximately 1 in 8 males and 1 in 10 females would develop cancer in Mbarara district before the age of 75 years. As indicated in Table 3, the risk (cumulative rate) of developing cancer in females increases from 6.8% in those aged 0–64 years to 9.7% in those aged 0–74 years and from 5.9 to 12.6% in males respectively.

### Availability of cancer risk factor data in the hospital records

The results of this study show that surveillance of cancer risk factors using hospital records is almost impossible in Uganda; as data on cancer-related risk factors was usually not available from the medical records. Missing data on cancer risk factors ranged from 65% for HIV to 99.8% for overweight and healthy feeding (Table 4). On average 86.5% of the cases had no documented information about the presence or absence of cancer risk factors in their records.

**Table 4:** Proportion of missing data for cancer risk factors in medical records

Variable	% Missing (n), N = 593
Tobacco use	91.7 (544)
Alcohol use	92 (545)
Physical inactivity	99.7 (591)
Health feeding	99.8 (592)
Overweight	99.8 (592)
History of cancer in a family	96.1 (570)
Hep B/C	99.7 (591)
HPV	99.7 (591)
H. Pylori	99.7 (591)
HIV	65.9 (391)
Age at Menarche	93.7 (556)
No. of children	81.9 (485)
Age at first full term pregnancy	93.4 (554)
History of breast feeding	98 (581)

### Quality of data

Overall, the quality of data was relatively good. Only 0.2% (3 cases) had missing age. There were no cases missing other mandatory variables (that is, patient names, usual residential address, sex, incident date; most valid basis of diagnosis; primary site; histological type and behaviour) including primary site uncertain (PSU percentage was 0%). The percentage of morphologically verified cases (MV%) for all cancers was 65.4%; this meets the expected standard value of MV% (61.1%) for sub-Saharan Africa, indicating accuracy of diagnosis [15].

***Proportion of cases with missing data***: Table 5 provides details of the missing variables (as per the interim analysis) and the reasons for missing variables. The mandatory variables for cancer surveillance that were recorded for each case are: patient names; usual residential address; age; sex; incident date; most valid basis of diagnosis; primary site; histological type; and behavior of the tumour. Among these, only age had some missing data for 12 cases (2%) during the interim analysis and for only 3 cases (0.2%) finally. However, non-mandatory variables, such as stage of cancer; sites of metastases; and laterality, had a high percentage of missing data, ranging from 79 to 99%. This is because these variables do not apply to all tumours and were always recorded as missing in cases where they did not apply.

***Re-abstracting Audit***: The overall percentage agreement between the two data abstractors was 71.3%. The agreement between the reviewers ranged from 35% for incident date to 92.5% for sex. Table 5 provides details of the agreement proportions for the specific variables with reasons for disagreement.

**Table 5:** Proportion of cases with missing variables and proportion of agreement among reviewers

Variable	% Missing (n)N = 593	% agreement (n) N = 80	Cause of discrepancy
Age	2 (12)	83.8 (65)	Age was missing in some primary sources that indicated adult instead of actual age.
Sex	0 (0)	92.5 (74)	The horizontal layout of the check boxes on the DAF caused some confusion.
Tribe (Ethnic group)	0 (0)	81.3 (65)	The responses were congested with so many checkboxes, some arranged horizontally.
Occupation	26.5 (157)	-	Not referring to the international classification of occupation manual.
Incident date	0 (0)	35 (28)	Differences in the hierarchy of determining the date of incidence.
Basis of diagnosis	0 (0)	72.5 (58)	Not updating basis of diagnosis when pathology reports are identified.
Site of Primary (Topography )	0 (0)	75 (60)	Variations were due to recording cancers as grouped vs. specific cancer site.
Morphology	0 (0)	83.8 (67)	Depended on used basis of diagnosis, that differed based on the source used.
TNM Stage	86.3 (512)	58.8 (47)	Some cancers are not staged using TNM staging.
Laterality	79.6 (472)	-	Similar to the TNM staging; not all organs are paired.
Sites of metastases	98.6 (585)	-	This was rarely captured in the primary source.
Treatment received	2.2 (13)	-	Not captured in the primary source
Date of last contact	2.7 (16)	53.8 (43)	The discrepancy was mainly due to inattention to detail.
Source of information	9. 3 (55)	-	Lack of attention to detail.

DAF = Data abstraction form; GIT = Gastrointestinal cancers; TNM = tumour, nodes and metastases

#### Morphologically verified case diagnosis

The proportion of cases with morphologically verified diagnosis (MV %) refers to the percentage of cases for which diagnosis is based on histology or cytology. A high MV% is taken to mean accuracy of diagnosis, whereas a low MV% casts doubt on the validity of the data. The MV% for all cancers from this study was 65.4%, Table 6; this meets the expected/standard value of MV% (61.1%) for sub-Saharan Africa, indicating accuracy of diagnosis [19].

**Table 6:** Proportion of cases with morphologically verified diagnosis

Basis of diagnosis (n)	No. of cases (N = 1258)	Percentage
Post mortem only	1	0.08%
Clinical diagnosis	434	34.5%
Clinical only (340)		
Clinical Investigations (91)		
Specific tumour markers (3)		
Morphological diagnosis	823	65.4%
Cytology/Haematology (38)		
Histology of metastases (10)		
Histology of primary tumour (775)		

### Resources needed to conduct the study

The funds spent on this study totaled £ 24,268.51 GBP. Table 7 provides a breakdown of the costs involved in conducting the study by major categories. Because economic feasibility was not among the primary objectives of the study, resources that were provided in kind were not costed/tracked, for example office space, water/electricity bills, internet costs, Information Technology (IT) support, and the vehicles used to transport staff to different data sources. These were provided by the researcher’s organization. Also, the time spent at each data source and time needed to abstract the data was not formally recorded, but varied depending on the context/setting. For example, facilities and records’ offices/departments that had a small number of, and/or well organized records and documentation systems demanded less time compared to others. Also the speed of data abstraction increased with time spent at the facility and as abstractors became familiar with the documentation practices/systems at a particular facility. The emphasis was on the quality of recorded data (accuracy and completeness) rather than the quantity (number of records abstracted per hour/day).

**Table 7:** Costs involved in conducting the feasibility study

Item	Cost (GBP)
Ethical clearance and Administrative costs	
REC initial review	310
REC annual continuing review	77
UNCST initial review/registration	38
Administrative clearance costs	950
Administrative clearance/approval from data sources (travel and accommodation)	2672
Communication (cell phone credit)	600
Air travel for the PhD student	1600
Sub total	**6247**
Equipment	
Study computer	770
Filling Cabinet	270.7
Box Files	146.2
**Sub total**	**1186.9**
Personnel and running Costs	
Administrative Assistant	1600
Data Entrant	3340.41
Study coordinator	3735
Finance Assistant	1200
**Sub total**	**9875.41**
Training of study staff	
Stationery	60
Day compensation	115
Facilitators	73
Per-diem and travel costs for upcountry (GCR) staff	108
Training Venue hire including refreshments	33.3
**Subtotal**	**389.3**
Data collection	
Stationary	186.3
Per-diem for the data collectors	2950
Per-diem for the driver	450
Fuel	369
Records retrieval	1500
Day allowance for district Surveillance officer	180.2
Day allowance for Health facility Record officers	240
Data collection in sources within Kampala	510.4
Driver for Kampala sources	184
**Sub total**	**6,569.9**
**Grand total**	**£ 24,268.51**

## Discussion

The aim of this study was to test the operational feasibility of conducting periodic studies, using a catchment population approach, to assess and monitor cancer incidence in areas not covered by PBCRs in Uganda. The results of this study indicate that estimating cancer incidence, using a retrospective cohort design and a “catchment population approach” is feasible in Uganda. This involves active registration of newly diagnosed cancer cases for a defined period of time, in all the health facilities serving a defined population [11]. The feasibility of these methods is demonstrated by the high accessibility and acceptability rates of the data sources in providing cancer data. The data sources were also supportive in navigating the patients’ records during the data collection process. Although many health workers were not knowledgeable about cancer registration, they were very enthusiastic to provide the data when its importance to cancer control was explained. This suggests high feasibility of such studies in the future. In addition, data on mandatory variables for cancer surveillance and estimating cancer incidence was available in the medical records and the quality of data was relatively good. This is in line with a national survey conducted in Malawi, in 2010, that found active case finding to be more robust in providing national cancer data than previously reported in the country [20].

On the question of data quality, case ascertainment and verification from this study meets the expected standard for sub-Saharan Africa (MV% of 65%), indicating sufficient accuracy of diagnosis based on regional standards. Moreover, the proportion of missing variables for all the mandatory variables was also within the acceptable standards (< 20%) for the region [15]. This implies that the available data sources and information are sufficient to provide the needed evidence for cancer control planning and implementation in Uganda. Data quality could be improved over time by designing less complex data collection tools, improving documentation and good clinical practices in clinical settings, and standardisation of pathology and cancer diagnostic guidelines in the country [21].

Although this study was carried out in one district only, its feasibility implies that such studies could be scaled up to assess cancer incidence at regional levels. This could contribute to national estimates and the assessment of geographical variation in cancer occurrence across the regions. In addition, the results of this study indicate the feasibility of evaluating targeted interventions at the population level, using interrupted time series, to ensure effective implementation of evidence based cancer control strategies [22, 23]. It is documented that such studies are vital for any setting, as there is always a need to retrospectively evaluate interventions that have been implemented at the population level, without randomization or control populations [22]. In addition, translating cancer epidemiological knowledge and methods into cancer control requires implementation of studies like this one at population level [24].

Another important aspect of this study was to assess the availability of cancer data to estimate cancer incidence. This study established that all the mandatory variables for estimating cancer incidence were available in the medical records and the estimated cancer incidence rates in Mbarara district were comparable with data from upcountry PBCRs in Uganda. Compared with the Gulu (in northern Uganda) cancer registry analysis for 2013 to 2016 period, Mbarara district shows a slightly higher burden of cancer, for all cancers combined in men, than in Gulu, whereas in females, Gulu indicates a slightly higher incidence. The cancer pattern is also slightly different: Mbarara has higher rates of cancers of affluence such as: prostate, oesophagus, and stomach, while Gulu has higher rates of infection-related cancers including: cancer of the cervix; liver; Non-Hodgkin lymphoma (NHL); and Kaposi’s sarcoma. These variations in cancer occurrence could be partly explained by the differences in socioeconomic status in the country. Gulu has higher levels of socio-economic deprivation and is probably exposed to more infections than Mbarara district [25–30]. Another possible explanation could be the difference in HIV prevalence rates, which are higher in Gulu (7.2%) than in Mbarara (5.7) [31]. This may explain the higher rates of HIV-associated cancers (cervix, Kaposi’s sarcoma, and NHL) in Gulu compared to Mbarara district.

The overall age-standardised incidence rates observed in Mbarara district are lower than the 2018 GLOBOCAN estimates for Uganda (overall ASIRs of 151.7 per 100,000 among males and 154.5 per 100,000 among females) and below those observed by Kampala population-based cancer registry (2011–2013 rates of 162.1 and 182 per 100,000 among males and females respectively) [7, 32, 33]. This could be explained by the differences in the two settings: Mbarara district is more rural than Kampala capital city (whose data GLOBOCAN estimates use) and may exhibit huge differences not only in the diagnostic/treatment infrastructure but also in the exposure to cancer risk factors and demography. Hence, these variations have to be investigated further given the fact that the estimated cancer incidence rates in Mbarara district were comparable with data from Gulu cancer registry (a similar upcountry PBCR) in Uganda.

Consistent with earlier studies, Mbarara district has higher rates of stomach cancer than Kampala for both males and females, and stomach cancer is among the top-five cancers in Mbarara, while it does not appear in the top-five cancers in Kampala [32]. The higher incidence of stomach cancer in Mbarara district has been previously documented by a hospital-based study conducted in 2002 [34]. Studies like this one, therefore, could allow regional comparisons and provide a platform for further investigations and possible explanations for the observed variations in cancer burden. Evidence that is locally generated, is very valuable in understanding the causes of cancer in different parts of the country. It also allows targeted and evidence-based cancer control strategies among different regions of the country.

The most obvious finding that emerged from this study was the inadequate availability of information on cancer risk factors in the medical records. As a result, this study was not able to assess prevalence of cancer risk factors among the registered cancer cases because this information was not always available in the medical records. This finding is in line with the continuously documented lack of socio-economic and demographic variables, as well as risk and behavioural-factor-related variables in medical records [35]. The practical implication for all health workers is the need to improve data capture about cancer risk factors for all patients to support cancer risk factor surveillance in the country. This also calls for qualitative assessment of the barriers and facilitators to documenting cancer risk factors in Uganda, and exploration of approaches to improving systematic recording, in health facilities, of cancer and other non-communicable diseases risk factors.

### Limitations and strengths of the study

This study is based on a retrospective records review and limitations in data quality could not be fully controlled. For example, some variables such as stage of disease were not available; while others, like age, were in some instances conflicting (for example, the laboratory report would indicate a different age or birth date or month from the radiology reports). Measurement error and data abstraction bias were minimized by use of standardized data collection forms and standard operating procedures (SOPs), with precise variable definitions and quality control and assurance measures.

Furthermore, it is possible that the incidence rates in this study were underestimated due to limitations in cancer diagnostic facilities in this setting and the exclusion of four important data sources for population based cancer registration data. In addition, the study did not track Death Certificate Only (DCO) cases due to the inadequate Death registration and Certification system in the country. Improved cancer diagnostics and information for vital status, death certificates and cause of death are vital in ensuring quality and completeness of cancer registration data. Although mortuary departments were assessed (and registered one case diagnosed at autopsy), the study failed to access the vital statistics data from the birth and death registration office at the National Identification and Regulatory Office (NIRA). This is because of the legislation pertaining to the confidentiality of death certificates, that it required detailed and lengthy negotiations with the relevant authorities. Negotiations to have access to identifiable death notification data by population-based cancer registries were started as a result of this study, but were not completed by the end of the data collection process. However, generally mortality data in Uganda is patchy because a significant proportion of deaths and burials occur at home and are rarely reported to municipal councils. In addition, autopsies are not always conducted and specific causes of death are not reported or known. Hence, these results provide an indication of the minimum cancer incidence rates in this population. Similarly, misclassification of cases that were not morphologically verified (i.e. those based on clinical diagnosis) could have occurred, but the high percentage (65%) of morphologically verified cases in this study provides some confidence that these results provide better estimates of cancer incidence rates in this population than not having any available statistics.

Another limitation of the study was the inability to comprehensively assess the economic feasibility and resources involved. For example, the study did not cost resources provided in kind; did not track time spent during data abstraction; and never conducted actual discussions of the realistic personnel salaries. This implies that future and larger studies may require more resources.

Although the completeness of data in Uganda needs to be improved (according to international quality indicators related to overall health-care infrastructure), one of the most significant strengths of this study is the ability to estimate cancer incidence using a catchment population approach, with good quality data. The morphological verification (MV%) of 65% meets and exceeds the predicted value and standards for Sub-Saharan Africa (SSA), which were set at 61.1% by Cancer Incidence in Five Continents (C15), volume VIII. This demonstrates the overall accuracy and validity of the data acquired in this study. Additionally, the study’s data sources were highly accessible and acceptable for providing information on cancer, and all the mandatory variables for estimating cancer incidence were available in the medical records. This shows that such studies are feasible and highlight a great opportunity for major improvements of cancer surveillance system in resource limited settings.

## Conclusion

The results of this study indicate that estimating cancer incidence, using a retrospective cohort design and a “catchment population approach” (named CORU method) is feasible in Uganda. Periodic population studies using this approach are potentially a precious resource for cancer surveillance research in resource-limited settings, where population-based cancer registries are still scarce. This could provide detailed and timely information to assess variations in cancer occurrence among different regions of the country and provide a more comprehensive picture of the cancer burden over time, in order to inform and direct cancer control policies in the country. The methods used for this study may be applied to other resource limited settings with no population-based cancer registries.

## Data Availability

The datasets used and/or analysed during the current study are available from the corresponding author on reasonable request.
